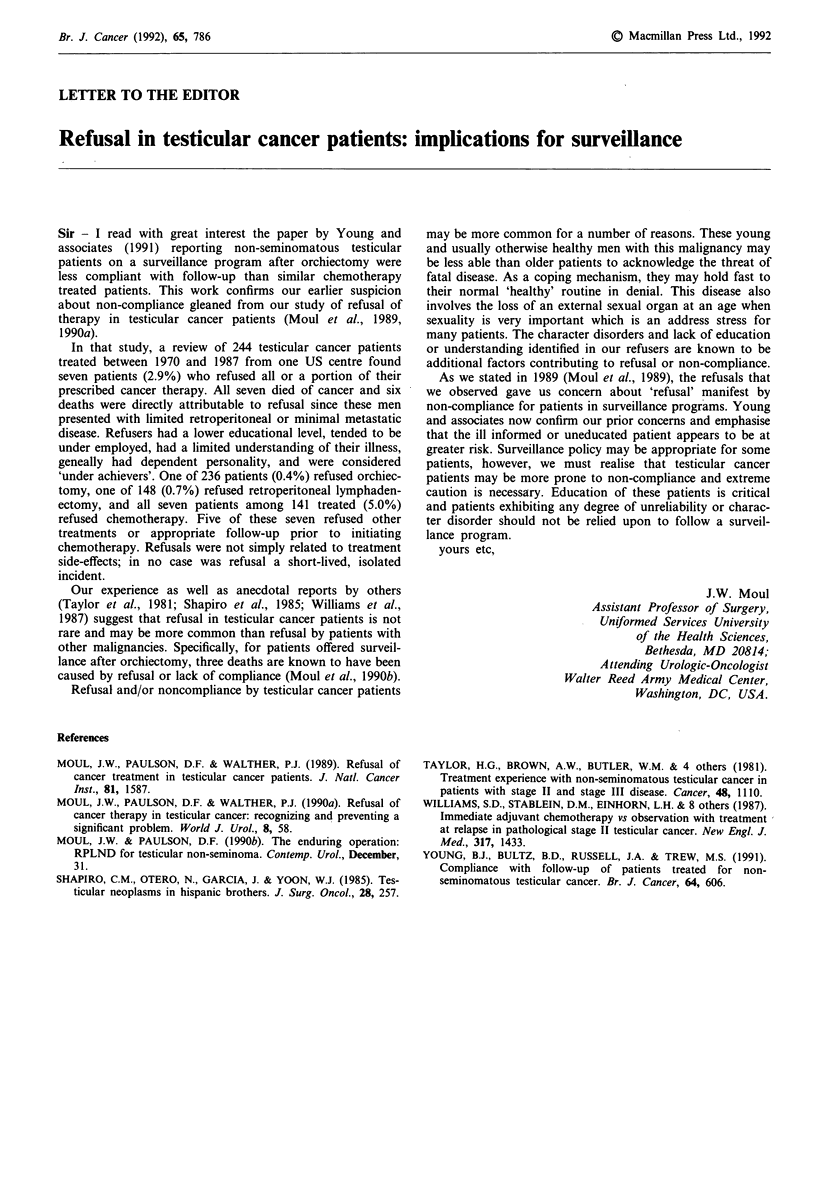# Refusal in testicular cancer patients: implications for surveillance.

**DOI:** 10.1038/bjc.1992.168

**Published:** 1992-05

**Authors:** J. W. Moul


 © Macmillan Press Ltd., 1992

LETTER TO THE EDITOR

### Refusal in testicular cancer patients: implications for surveillance

**Sir** – I read with great interest the paper by Young and associates (1991) reporting non-seminomatous testicular patients on a surveillance program after orchiectomy were less compliant with follow-up than similar chemotherapy treated patients. This work confirms our earlier suspicion about non-compliance gleaned from our study of refusal of therapy in testicular cancer patients (Moul *et al.*, 1989, 1990*a*).

In that study, a review of 244 testicular cancer patients treated between 1970 and 1987 from one US centre found seven patients (2.9%) who refused all or a portion of their prescribed cancer therapy. All seven died of cancer and six deaths were directly attributable to refusal since these men presented with limited retroperitoneal or minimal metastatic disease. Refusers had a lower educational level, tended to be under employed, had a limited understanding of their illness, geneally had dependent personality, and were considered 'under achievers'. One of 236 patients (0.4%) refused orchiectomy, one of 148 (0.7%) refused retroperitoneal lymphadenectomy, and all seven patients among 141 treated (5.0%) refused chemotherapy. Five of these seven refused other treatments or appropriate follow-up prior to initiating chemotherapy. Refusals were not simply related to treatment side-effects; in no case was refusal a short-lived, isolated incident.

Our experience as well as anecdotal reports by others (Taylor *et al.*, 1981; Shapiro *et al.*, 1985; Williams *et al.*, 1987) suggest that refusal in testicular cancer patients is not rare and may be more common than refusal by patients with other malignancies. Specifically, for patients offered surveillance after orchiectomy, three deaths are known to have been caused by refusal or lack of compliance (Moul *et al.*, 1990*b*).

Refusal and/or noncompliance by testicular cancer patients may be more common for a number of reasons. These young and usually otherwise healthy men with this malignancy may be less able than older patients to acknowledge the threat of fatal disease. As a coping mechanism, they may hold fast to their normal 'healthy' routine in denial. This disease also involves the loss of an external sexual organ at an age when sexuality is very important which is an address stress for many patients. The character disorders and lack of education or understanding identified in our refusers are known to be additional factors contributing to refusal or non-compliance.

As we stated in 1989 (Moul *et al.*, 1989), the refusals that we observed gave us concern about 'refusal' manifest by non-compliance for patients in surveillance programs. Young and associates now confirm our prior concerns and emphasise that the ill informed or uneducated patient appears to be at greater risk. Surveillance policy may be appropriate for some patients, however, we must realise that testicular cancer patients may be more prone to non-compliance and extreme caution is necessary. Education of these patients is critical and patients exhibiting any degree of unreliability or character disorder should not be relied upon to follow a surveillance program.

yours etc,

J.W. Moul
*Assistant Professor of Surgery,*
*Uniformed Services University*
*of the Health Sciences,*
*Bethesda, MD 20814;*
*Attending Urologic-Oncologist*
*Walter Reed Army Medical Center,*
*Washington, DC, USA.*

**References**

MOUL, J.W., PAULSON, D.F. & WALTHER, P.J. (1989). Refusal of cancer treatment in testicular cancer patients. *J. Natl. Cancer Inst.*, **81**, 1587.

MOUL, J.W., PAULSON, D.F. & WALTHER, P.J. (1990*a*). Refusal of cancer therapy in testicular cancer: recognizing and preventing a significant problem. *World J. Urol.*, **8**, 58.

MOUL, J.W. & PAULSON, D.F. (1990*b*). The enduring operation: RPLND for testicular non-seminoma. *Contemp. Urol.*, **December**, 31.

SHAPIRO, C.M., OTERO, N., GARCIA, J. & YOON, W.J. (1985). Testicular neoplasms in hispanic brothers. *J. Surg. Oncol.*, **28**, 257.

TAYLOR, H.G., BROWN, A.W., BUTLER, W.M. & 4 others (1981). Treatment experience with non-seminomatous testicular cancer in patients with stage II and stage III disease. *Cancer*, **48**, 1110.

WILLIAMS, S.D., STABLEIN, D.M., EINHORN, L.H. & 8 others (1987). Immediate adjuvant chemotherapy *vs* observation with treatment at relapse in pathological stage II testicular cancer. *New Engl. J. Med.*, **317**, 1433.

YOUNG, B.J., BULTZ, B.D., RUSSELL, J.A. & TREW, M.S. (1991). Compliance with follow-up of patients treated for non-seminomatous testicular cancer. *Br. J. Cancer*, **64**, 606.